# CITOBOT AI for real-world cervical cancer screening using colposcopy imaging

**DOI:** 10.3389/fpubh.2026.1802494

**Published:** 2026-06-12

**Authors:** Marcela Arrivillaga, Daniela Neira, David Steven Rivero, Hernán Dario Vargas-Cardona, Paula C. Bermúdez, Juan Pablo García-Cifuentes, Juan Carlos Aristizabal, Maria del Mar Torres, Andres Jaramillo-Botero, Diana Maria Castrillón

**Affiliations:** 1 Department of Public Health and Epidemiology, Pontificia Universidad Javeriana, Cali, Colombia; 2 Alcaldía de Santiago de Cali, Red de Salud Ladera ESE, Cali, Colombia; 3 Department of Electronics and Computer Science, Pontificia Universidad Javeriana, Cali, Colombia; 4 Department of Maternal and Child Health, Pontificia Universidad Javeriana, Cali, Colombia; 5 Institute of Omics Sciences, Pontificia Universidad Javeriana, Cali, Colombia

**Keywords:** artificial intelligence in public health, cervical cancer screening, colposcopy imaging, deep learning models, digital health

## Abstract

**Background:**

Cervical cancer remains a major cause of morbidity and mortality among women, particularly in low- and middle-income countries, where delays in screening and diagnostic follow-up limit early detection. Artificial intelligence applied to medical imaging may strengthen screening programs by improving accuracy, consistency, and timeliness in resource-limited settings.

**Objective:**

This study aimed to evaluate the internally validated screening performance of CITOBOT AI, an artificial intelligence system for real-world cervical cancer screening using colposcopy imaging, in a public hospital setting in Colombia.

**Methods:**

A cross-sectional study was conducted among 650 women screened at ‘Siloé’ Hospital in Cali, Colombia, between February 2023 and July 2025. Colposcopy-guided biopsy served as the reference standard. A total of 2,648 cervical images were classified using a predefined binary endpoint: screen-negative/no risk versus screen-positive/at risk. The model was developed using transfer learning, image segmentation, data augmentation, patient-level data partitioning, and five-fold cross-validation within the training subset. Screening performance was evaluated using accuracy, sensitivity, specificity, area under the receiver operating characteristic curve, predictive values, and confusion matrix analysis. A secondary exploratory analysis examined associations between selected clinical variables and AI-based screening classification.

**Results:**

CITOBOT AI achieved internally validated screening performance with an accuracy of 94.3%, sensitivity of 93.4%, specificity of 94.9%, and an area under the receiver operating characteristic curve of 0.98. Positive and negative predictive values were 92.9 and 96.2%, respectively. Performance estimates were stable across patient-level folds and consistent with the hold-out validation subset within the same dataset. HPV status showed an unexpected inverse association with AI screen-positive classification; this finding should not be interpreted as biologically protective or as evidence that HPV status influenced model output, since HPV status was not used as an input variable.

**Conclusion:**

CITOBOT AI demonstrated promising internally validated performance for real-world cervical cancer screening based on colposcopy imaging. The predefined binary classification is consistent with its intended role as a screening support tool to identify women who may require confirmatory colposcopy and biopsy, while histopathology remains the reference standard for lesion grading and therapeutic decision-making. External validation in independent multicenter and population-based datasets is required before broader implementation.

## Introduction

Cervical cancer remains a major global public health challenge and a leading threat to women’s health worldwide. Although it is entirely preventable when detected early, it continues to cause substantial mortality due to persistent gaps in timely screening and intervention. Early detection is critical, as it significantly improves survival and quality of life. According to GLOBOCAN 2022, cervical cancer has a global mortality rate of 7.1 per 100,000, accounting for 348,874 deaths and ranking as the ninth leading cause of cancer-related mortality worldwide. The global incidence rate is 14.1 per 100,000, with the highest burden concentrated in Asia, Africa, and Latin America and the Caribbean. Notably, mortality in Latin America and the Caribbean is four times higher than in the United States, highlighting profound inequities in access to preventive and diagnostic healthcare.

Current cervical cancer prevention strategies include human papillomavirus (HPV) vaccination (1), cervical cytology screening (Pap smears) (2), HPV DNA testing (3), and visual inspection with acetic acid or Lugol’s iodine (VIA/VILI) (4, 5). When indicated, colposcopy and biopsy are employed as confirmatory diagnostic procedures. Despite the availability of these interventions, global coverage of cervical cancer screening remains highly uneven, and adherence continues to be a critical determinant of screening effectiveness. In 2019, the global adherence rate to cervical cancer screening was approximately 33.66%, with a pronounced disparity between high-income countries (75.66%) and low- and middle-income countries (24.91%) (6), reflecting persistent structural, economic, and health system barriers.

Recent studies have increasingly highlighted the role of artificial intelligence (AI) and machine learning (ML) in gynecologic oncology, with applications in cancer prediction, screening, detection, prognosis, treatment selection, and clinical decision support (7–9). In endometrial cancer, for example, ML approaches have been reviewed as tools for prevention, screening, detection, and prognostication, illustrating the broader relevance of computational methods across gynecologic malignancies (8). Similarly, recent reviews in precision oncology have emphasized the integration of bioinformatics, multiomics, AI, and ML tools to improve biomarker discovery, predictive modeling, diagnostic accuracy, and personalized treatment strategies (9). Within cervical cancer specifically, a systematic analysis demonstrated the effectiveness of MRI-based radiomic models for predicting preoperative lymphovascular invasion, achieving a sensitivity of 83% and a specificity of 74%, supporting their potential as non-invasive diagnostic tools (10). In parallel, reviews of AI-based approaches for automated cervical cancer detection have identified deep learning models as particularly effective for medical image analysis, while emphasizing the need for larger and more diverse datasets to ensure model robustness and generalizability (11). Beyond MRI and cytology, AI applications in ultrasonography have shown high accuracy in patient stratification and histopathological correlation, particularly in distinguishing benign from malignant lesions (12).

Importantly, studies conducted in low-resource settings have explored the feasibility and acceptability of AI-driven screening tools. In Cameroon, smartphone-based AI applications were generally considered acceptable, although concerns related to privacy, data security, and trust were reported, underscoring the importance of confidentiality safeguards and user education (13). Similarly, in China, an AI-assisted cytology system improved the detection of precancerous lesions in high-risk populations, highlighting the potential of AI to strengthen screening programs in contexts with limited vaccination coverage and suboptimal screening uptake (14). However, despite these advances, evidence remains limited regarding the use of AI systems based on colposcopy imaging within real-world, population-based screening programs, particularly in low- and middle-income countries.

A significant barrier to screening adherence, frequently cited in the literature, is the delay in cytology result delivery, which can have serious consequences for both patients and healthcare systems (15–18). Such delays may contribute to the progression of precancerous lesions to invasive cancer, diagnoses at more advanced stages, emotional distress, reduced motivation for future screenings, misinterpretation of results, and delays in treatment initiation. Collectively, these factors may necessitate more aggressive treatments, increase healthcare costs, strain healthcare resources, and ultimately undermine trust in the healthcare system.

To address these delays and improve the effectiveness of cervical cancer screening, advances in deep learning–based image analysis have been increasingly applied to cervical cancer risk stratification. Convolutional neural networks and hybrid architectures, combined with strategies such as transfer learning and data augmentation, allow pre-trained models to be adapted to cervical image classification tasks, improving performance, generalizability, and reproducibility even when training datasets are relatively limited (19–21). Recent integrations of convolutional neural networks with vision transformers have further strengthened image-based risk classification, supporting scalable and robust screening applications.

In parallel, some studies have explored the integration of multimodal data—such as colposcopy images and clinical information—to support more comprehensive risk assessment and personalized screening recommendations. While these approaches may enhance clinical decision-making, they also introduce additional data dependencies and operational complexity that may limit scalability and feasibility in population-based screening programs, particularly in resource-constrained settings.

Building on these technological advances and responding to the need for context-appropriate innovations to strengthen population-based cervical cancer screening as a preventive strategy, this study aimed to evaluate the screening performance of CITOBOT AI, an artificial intelligence system developed for real-world cervical cancer screening using colposcopy imaging. Specifically, the study assessed the ability of CITOBOT AI to classify women as screen-negative or screen-positive in real-world clinical screening settings, with relevance for improving coverage, timeliness, and decision-making in resource-limited contexts in Latin America.

## Materials and methods

### Study design

This cross-sectional study was conducted to evaluate the screening performance of artificial intelligence algorithms for cervical cancer risk classification, with emphasis on sensitivity and specificity. The study was approved by the Ethics Committee of the Faculty of Health Sciences at Pontificia Universidad Javeriana Cali, Colombia (Approval No. 002-2022; April 20, 2022), and was carried out in accordance with the principles of the Declaration of Helsinki and applicable national regulations governing biomedical research involving human participants.

Written informed consent was obtained from all participants prior to enrollment, both for participation in the study and for the publication of anonymized data, following a comprehensive explanation of the study objectives and procedures. Participant confidentiality was safeguarded through data anonymization, and all individuals retained the right to withdraw from the study at any time without any consequences.

### Participants

A total of 650 women were recruited from ‘Siloé’ Hospital in Cali, Colombia, between February 2023 and July 2025. Eligibility criteria included being of reproductive age, having undergone colposcopy and biopsy within the previous 2 months, and having no history of prior surgical treatment of the cervix. Unlike traditional case–control designs, this cross-sectional study enrolled all eligible participants without predefined grouping, enabling a comprehensive assessment of the AI algorithm’s screening performance across a broad spectrum of clinical outcomes. Colposcopy and histopathological biopsy results served as the reference standard for confirming the presence or absence of cervical lesions.

### Sample size

The sample size was calculated using the Hajian-Tilaki method (22) for diagnostic accuracy studies, ensuring adequate representation of both positive and negative biopsy outcomes. The calculation was based on estimation of sensitivity and specificity for a binary screening test, assuming a statistical power of 80% and a two-sided significance level of 0.05. The expected distribution of biopsy-confirmed positive and negative cases was considered to ensure sufficient precision for the primary screening performance estimates. The final sample of 650 participants provided adequate representation of both screening categories for internal validation of the CITOBOT AI model.

### Data collection

Data were collected using structured questionnaires and medical record review. The information obtained included demographic characteristics, relevant clinical history, and established cervical cancer risk factors.

Cytology results were extracted from medical records and classified according to the Bethesda System into the following categories: Negative for Intraepithelial Lesion or Malignancy (NILM), Low-Grade Squamous Intraepithelial Lesion (LSIL), Atypical Squamous Cells of Undetermined Significance (ASC-US), Atypical Squamous Cells—cannot exclude HSIL (ASC-H), High-Grade Squamous Intraepithelial Lesion (HSIL), endocervical adenocarcinoma *in situ* (AIS), endocervical adenocarcinoma, other findings, or not reported.

Colposcopic findings were obtained from standardized colposcopy reports and categorized as cancer, High-Grade Squamous Intraepithelial Lesion (HSIL), Low-Grade Squamous Intraepithelial Lesion (LSIL), negative for lesion, inflammation, metaplasia, dystrophy, ulcer, or other findings.

Biopsy results served as the diagnostic reference standard. Histopathological findings were classified as normal, cervical intraepithelial neoplasia grade I (CIN I), cervical intraepithelial neoplasia grade II (CIN II), cervical intraepithelial neoplasia grade III/carcinoma *in situ* (CIN III/CIS), or carcinoma/adenocarcinoma.

### Laboratory analysis

Histopathological evaluation of biopsy specimens was performed at SynLab Laboratory by certified pathologists, following standardized procedures. Tissue processing included staining and detailed microscopic examination to confirm or exclude cervical abnormalities. Pathologists were blinded to the AI algorithm’s predictions to minimize observer bias.

### CITOBOT AI model development and validation

The development of the CITOBOT AI system followed a structured, multi-stage process designed to ensure robustness, reproducibility, and clinical relevance.

1. Exploration and experimentation. The initial phase involved exploratory analyses and experimentation using a database of 1,310 cervical images obtained with authorization from the National Cancer Institute (USA) and the World Health Organization. This phase focused on evaluating different deep learning architectures to optimize performance while accounting for the characteristics and limitations of cervical colposcopy images.
  During this stage, the need for image segmentation emerged as a critical requirement to reduce background noise and focus model attention exclusively on clinically relevant cervical regions. Implementing segmentation significantly improved input data quality and resulted in preliminary models with promising performance metrics.
2. Image dataset curation and labeling. Study-specific data collection yielded a dataset comprising 2,648 colposcopy images from 650 patients. All images were independently reviewed, classified, and labeled by cervical pathology experts into two predefined screening categories: No Risk, corresponding to lesion-free images, and At Risk, including cervical intraepithelial neoplasia grades I–III (CIN I–III) and invasive cancer. This expert-driven labeling process ensured high-quality reference annotations for model training and validation.
  The binary classification was defined according to the intended use of CITOBOT AI as a screening support tool rather than as a diagnostic or histopathological grading system. The purpose of the model is not to differentiate among CIN I, CIN II, CIN III, or invasive cancer, but to identify women who should be classified as screen-positive and referred for confirmatory evaluation through colposcopy and biopsy. Therefore, the At Risk category was designed to capture biopsy-confirmed cervical abnormalities that warrant confirmatory clinical assessment within the screening pathway.
  The mapping between histopathological reference categories and the predefined CITOBOT AI binary evaluation labels is shown in Table 1.
3. Data preparation and preprocessing. Images were processed in RGB format to preserve critical chromatic information relevant for automated classification. All images were acquired using a standard digital colposcope equipped with an integrated camera system, optical magnification, and dedicated illumination, under routine clinical conditions at the participating hospital. No images were excluded based on image quality, as all images were technically processable for segmentation and model evaluation.
  Preprocessing and segmentation algorithms were applied uniformly to all images to standardize model inputs, optimize computational efficiency, and focus the analysis on the cervical region of interest. Specific preprocessing steps included normalization, with pixel intensities scaled between 0 and 1, and data augmentation, including rotations, cropping, and lighting adjustments, to enrich the training dataset, reduce overfitting, and improve internal generalizability. To address class imbalance between categories, balancing strategies were implemented to ensure equitable representation and stable model performance across both classes. Image segmentation was performed using the Segment Anything Model (SAM), a general-purpose segmentation framework applied in this study to identify cervical regions of interest and reduce the influence of non-cervical background structures and acquisition-related artifacts without requiring extensive image-specific adjustments (23).
4. AI model design and training. Multiple deep learning architectures were evaluated, including AlexNet, GoogLeNet, U-Net, ResNet, VGGNet, and transformer-based models. Among these, InceptionV3 demonstrated superior performance for cervical image classification and was selected as the final architecture. Its design enables effective multi-scale feature extraction, capturing both low-level patterns (e.g., edges and textures) and higher-level structural features relevant to cervical pathology (24).
  Transfer learning was applied using InceptionV3 pre-trained on the ImageNet dataset (25), providing a robust initialization for feature extraction. Fine-tuning of the final layers allowed the model to adapt specifically to cervical image classification, optimizing performance with a moderate dataset size.
5. Model validation. The dataset was partitioned at the patient level to avoid data leakage arising from multiple images obtained from the same participant. Specifically, all images belonging to a given patient were assigned exclusively to a single partition and were not allowed to appear simultaneously in training and validation subsets. Patients were randomly allocated to training and hold-out validation subsets within the same dataset using an 80/20 split, after which all images from each patient followed the corresponding patient-level assignment. Model development and hyperparameter tuning were performed using the training subset only.
  A five-fold cross-validation strategy was then applied within the training subset using patient-level folds. In each fold, all images from the same patient were kept together, ensuring that no patient contributed images to both the training and fold-validation partitions. The hold-out validation subset within the same dataset was excluded from model training, hyperparameter tuning, and cross-validation, and was used only for the final evaluation of model performance on unseen patients from the same single-center clinical dataset. No external validation dataset was used in this study; therefore, all reported performance estimates should be interpreted as internally validated results derived from a single-center clinical dataset.
6. Model implementation and deployment. Model training and evaluation were conducted using Python with the TensorFlow and Keras libraries. Hyperparameter optimization was performed using grid search, while early stopping was applied to halt training when validation performance plateaued, thereby preventing overfitting. The learning rate was dynamically adjusted using the ReduceLROnPlateau callback, progressively decreasing from 10^−4^ to 10^−7^ to facilitate stable convergence.
  For real-world deployment, the finalized model was converted to TensorFlow Lite and integrated into a mobile application developed in Android Studio. This implementation enabled rapid, on-device risk classification within seconds, including offline functionality, supporting usability in resource-limited clinical screening settings.

**Table 1:** Mapping between histopathological reference categories and CITOBOT AI binary evaluation labels.

Histopathological reference category	CITOBOT AI binary evaluation label	Screening interpretation
Normal	Negative for lesions/no risk	Screen-negative
CIN I	At risk	Screen-positive
CIN II		
CIN III/carcinoma in situ		
Carcinoma/adenocarcinoma		

The binary mapping reflects the intended use of CITOBOT AI as a screening support tool. A screen-positive result indicates referral for confirmatory colposcopy and biopsy, while histopathology remains the reference standard for lesion grading and therapeutic decision-making.

### Data analysis

Statistical analyses were conducted using Stata version 15 (StataCorp, College Station, TX, USA). Descriptive statistics were used to summarize sociodemographic characteristics, clinical background, and diagnostic findings of the study population. Categorical variables were described using frequencies and percentages, while continuous variables were summarized using means and ranges, as appropriate.

The screening performance of CITOBOT AI was evaluated by comparing the AI-based risk classification (At Risk vs. Negative for lesions) with the clinical reference standard derived from colposcopy-guided biopsy. Performance metrics were estimated for the predefined binary endpoint: screen-negative versus screen-positive. This endpoint reflects the intended role of CITOBOT AI as a screening support tool, where a positive result guides referral for confirmatory colposcopy and biopsy rather than establishing lesion grade or treatment indication. Diagnostic accuracy metrics included accuracy, sensitivity, specificity, precision, positive predictive value, negative predictive value, confusion matrix, and the area under the receiver operating characteristic curve (AUC). These metrics were selected to assess internal classification and discriminative performance, rather than probabilistic risk calibration. Receiver operating characteristic curves were generated to evaluate discrimination across classification thresholds. Model performance was assessed using a patient-level 80/20 training–hold-out validation split within the same dataset and five-fold cross-validation applied only to the training subset. For both the initial split and the cross-validation folds, patient identifiers were used as grouping units so that all images from the same patient remained within the same partition. This procedure was implemented to prevent data leakage and to ensure that performance estimates reflected generalization to unseen patients within the same single-center dataset rather than recognition of correlated images from the same individual.

To explore relationships between selected non-visual clinical variables and AI-based screening classification, a secondary analysis was conducted. Univariable logistic regression models were first estimated to evaluate crude associations between selected demographic and clinical variables and the AI-based risk classification. Multivariable logistic regression models were then fitted by entering selected variables simultaneously. Variables were chosen *a priori* based on clinical relevance and availability in the study dataset, including age group, selected clinical antecedents, HPV status, and anthropometric indicators. No stepwise variable selection procedure was used. Odds ratios with 95% confidence intervals were reported. This analysis was not intended to establish clinical causality or statistical independence, but to explore whether AI-based screening classification was associated with selected non-visual clinical variables. Statistical significance was defined as a two-sided *p*-value < 0.05.

## Results

### Sociodemographic characteristics and clinical background

A total of 650 women participated in the study. Of these, 94.14% resided in Cali, Colombia. The mean age was 41 years (range: 18–74 years). Most participants had completed secondary education, belonged to a low socioeconomic stratum, were engaged in self-employment or informal occupations, and were enrolled in the subsidized health insurance regime. Additionally, 52.41% were married or living in a stable union. Detailed sociodemographic characteristics are presented in Table 2.

**Table 2:** Sociodemographic characteristics of women screened with the CITOBOT AI system (*n* = 650).

Characteristic	Frequency (*n*)	Percentage (%)
City of origin		
Cali	611	94.14
Other Colombian cities	8	1.23
Venezuelan migrants	30	4.62
Missing data	1	–
Educational level		
Primary education	138	21.46
Secondary education	432	67.19
Technical or vocational training	52	8.09
University education	17	2.64
No formal education	4	0.62
Missing data	7	–
Marital status		
Married/cohabiting	337	52.41
Single	206	32.04
Separated/widowed	70	10.89
No response	37	–
Occupation		
Employed	82	12.75
Unemployed	17	2.64
Homemaker	256	39.81
Self-employed	159	24.73
Informal worker	114	17.73
Student	11	1.71
Retired/pensioned	3	0.47
Does not know/no response	8	–
Socioeconomic stratum^*^		
Stratum 1 (lowest income)	534	83.83
Stratum 2	97	15.23
Stratum 3 and 4	6	0.94
Missing data	13	–
Health insurance regime^**^		
Contributory	14	2.19
Subsidized	605	94.68
Special (military, teachers)	3	0.47
Uninsured	16	2.51
Missing data	2	–

^*^Socioeconomic stratum refers to the official classification system used in Colombia to categorize households according to housing and neighborhood conditions, ranging from Stratum 1 (lowest income and living conditions) to Stratum 6 (highest income and living conditions). This classification is used for determining access to social subsidies and public service tariffs.

^**^Health insurance regime corresponds to the type of affiliation within Colombia’s General System of Social Security in Health. It includes:

Contributory regime: for individuals and families who contribute through formal employment or independent income;

Subsidized regime: for low-income populations whose health coverage is financed by the state;

Special regimes: for specific occupational groups such as the military or public school teachers;

Uninsured: individuals not affiliated with any regime.

Regarding clinical background and selected risk factors, hypertension was the most frequently reported chronic condition, affecting 11.13% of participants. A history of sexually transmitted infections was reported by 26.28% of women. Anthropometric assessment indicated that 64.89% of participants were classified as overweight according to World Health Organization body mass index criteria. Among participants with available abdominal circumference measurements, 31.31% met WHO criteria for central obesity. These clinical and anthropometric characteristics are summarized in Table 3.

**Table 3:** Clinical background and risk factors of participants (*n* = 650).

Variable		Category	Frequency (*n*)	Percentage (%)
Medical history *n* = 647				
History of hypertension		Yes	72	11.13
History of diabetes		Yes	37	5.72
History of cancer		Yes	25	3.86
Sexually transmitted infections (STIs)				
History of STI (general)		Yes	170	26.28
History of human papillomavirus (HPV) infection		Yes	160	24.73
History of syphilis		Yes	16	2.47
History of HIV infection		Yes	14	2.16
Lifestyle factor *n* = 645				
Smoker		Yes	22	3.41
Anthropometric indicators				
Body Mass Index (BMI)^*^		Underweight	18	2.82
		Normal weight	206	32.29
		Overweight	414	64.89
Abdominal circumference^**^	*n* = 329	Without risk	226	68.69
		At risk	103	31.31

^*^BMI categories follow WHO standards: underweight (<18.5 kg/m^2^), normal (18.5–24.9 kg/m^2^), overweight (25.0–29.9 kg/m^2^), and obesity (≥30 kg/m^2^).

^**^Abdominal circumference risk based on WHO cutoffs for central obesity (>88 cm for women).

### Clinical and diagnostic findings

Clinical and diagnostic findings revealed a wide distribution of cervical pathology within the study population. While a substantial proportion of participants presented with normal findings, others exhibited low-grade lesions, and a smaller subset showed high-grade abnormalities or histopathological confirmed carcinoma. The distribution of cervical pathology across diagnostic categories is presented in Table 4 and provides the clinical context for subsequent analyses of CITOBOT AI screening performance.

**Table 4:** Clinical and diagnostic results of participants (*n* = 650).

Test	Category	Frequency (*n*)	Percentage (%)
Cytology results	Negative for intraepithelial lesion or malignancy (NILM)	263	40.46
	Atypical Squamous Cells of Undetermined Significance (ASC-US)	80	12.31
	Low-Grade Squamous Intraepithelial Lesion (LSIL)	187	28.77
	Atypical Squamous Cells—cannot exclude HSIL (ASC-H)	6	0.92
	High-grade Squamous Intraepithelial Lesion (HSIL)	15	2.31
	Endocervical adenocarcinoma in situ (AIS)	15	2.31
	Endocervical adenocarcinoma	1	0.15
	Other findings	63	9.69
	Not reported	20	3.08
Colposcopic findings	High-grade abnormal findings		
	Cancer	4	0.62
	High-grade squamous intraepithelial lesion (HSIL)	41	6.33
	Low-Grade Squamous Intraepithelial Lesion (LSIL)	118	18.21
	Minor/non-specific changes		
	Atrophy	69	10.65
	Inflammation	208	32.1
	Dystrophy	24	3.7
	Ulcer	9	1.39
	Other findings	29	4.48
	Normal		
	Negative for lesion	145	22.38
	Not reported	1	0.15
	Missing data	2	–
Biopsy results	Normal	404	62.15
	Carcinoma	37	5.69
	Cervical intraepithelial neoplasia grade II (CIN II)	39	6.00
	Cervical intraepithelial neoplasia grade I (CIN I)	170	26.15

### CITOBOT AI

The CITOBOT AI system demonstrated high screening performance for cervical image classification within the internal validation framework, with consistent ability to distinguish between normal and at-risk cervical images. Performance metrics obtained from five-fold cross-validation were stable across patient-level folds and consistent with those observed in the hold-out validation subset within the same dataset. Because all images from each patient were restricted to a single partition, this approach reduced the risk of inflated performance due to image-level data leakage. (Table 5).

**Table 5:** Screening performance of the CITOBOT AI system for cervical image classification (*n* = 650).

Metric	Mean ± SD across folds^*^	Internal validation estimate, % (95% CI)^**^
Accuracy	94.30 ± 0.88	94.92 (92.92–96.34)
Sensitivity	93.39 ± 1.54	93.98 (90.29–96.31)
Specificity	94.91 ± 0.85	95.51 (92.98–97.13)
AUC (ROC Curve)	0.981 ± 0.007	–

^*^Values represent the mean ± standard deviation across five patient-level cross-validation folds. Accuracy, sensitivity, and specificity are reported as percentages; AUC is reported on a 0–1 scale.

^**^Internal validation estimates were calculated from the held-out patient-level internal validation subset. The 95% confidence intervals for accuracy, sensitivity, and specificity were calculated using Wilson binomial intervals.

AUC is reported as mean ± standard deviation across cross-validation folds; its confidence interval was not estimated because it requires predicted probabilities or continuous model scores rather than the confusion matrix.

All images from the same patient were restricted to a single partition to prevent data leakage.

**Figure 1:**
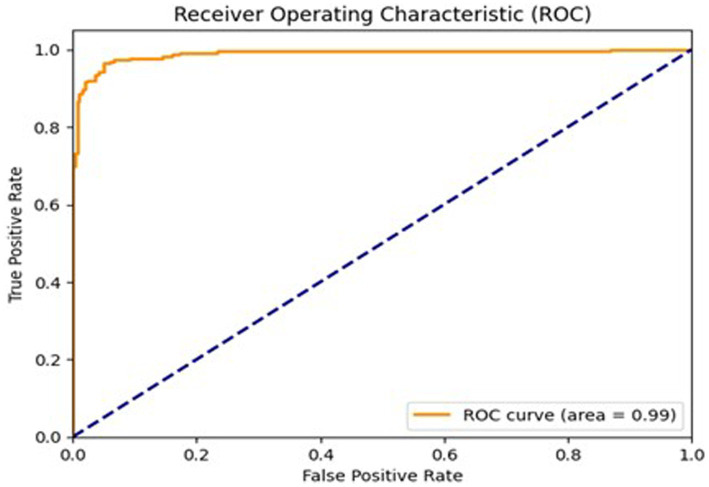
Receiver Operating Characteristic (ROC) curve for the CITOBOT AI screening model.

The Receiver Operating Characteristic (ROC) curve illustrates the discriminative performance of CITOBOT AI based on the internal validation data, showing separation between true positive and false positive rates across classification thresholds (Figure 1). This visual representation is consistent with the quantitative performance metrics reported in Table 5.

Category-specific performance metrics are presented in Table 6. For Category 0 (Negative for Lesions), precision, recall, and F1-score were 96.23, 95.51, and 95.86%, respectively. For Category 1 (At Risk), precision was 92.85%, recall was 93.98%, and the F1-score was 93.41%. The similarity of F1-scores across categories indicates comparable classification performance between normal and at-risk images.

**Table 6:** Category-specific metrics of CITOBOT AI.

Metric	Category 0 (negative for lesions)	Category 1 (at-risk)
Precision (%)	96.23	92.85
Recall (Sensitivity) (%)	95.51	93.98
F1-score (%)	95.86	93.41

The confusion matrix was constructed using the predefined binary screening labels from the curated AI evaluation dataset. Biopsy-confirmed normal findings were classified as Negative for lesions, while CIN I, CIN II, CIN III/CIS, and carcinoma/adenocarcinoma were classified as At Risk, as described in Table 1. Thus, Table 7 should be interpreted as a binary screening performance matrix rather than as a lesion-grade-specific diagnostic matrix or as a direct reproduction of the histopathological distribution in Table 4. Most cases were correctly classified, with 18 false positive and 15 false negative classifications. The model achieved positive and negative predictive values of 92.86 and 96.23%, respectively.

**Table 7:** Confusion matrix of the CITOBOT AI screening model (counts).

Observed category	Predicted: negative for lesions (Category 0)	Predicted: at risk (Category 1)	Total
No risk (Category 0)	383	18	401
At risk (Category 1)	15	234	249
Total	398	252	650

**Figure 2:**
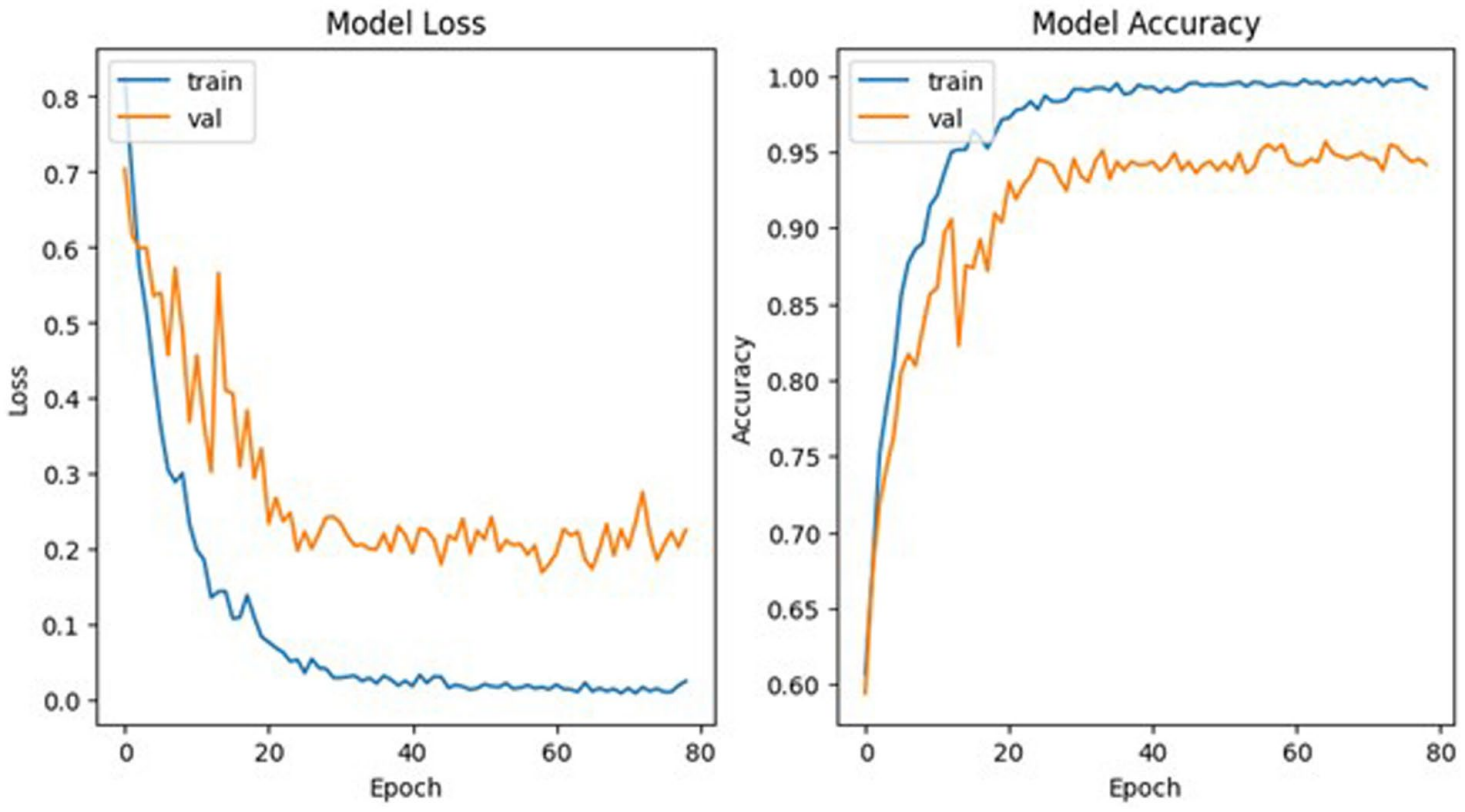
Training and validation performance of the CITOBOT AI model.

Training and validation performance during model development are shown in Figure 2. Accuracy and loss curves demonstrated stable convergence across epochs, with no evidence of divergence between training and validation trajectories.

### Secondary analysis: associations between clinical variables and AI-based screening classification

Following the evaluation of CITOBOT AI screening performance, a secondary analysis was conducted to examine the association between selected demographic and clinical variables and the AI-based screening classification. Univariable logistic regression analyses showed an association between age and screening outcome, with higher odds of being classified as screen-positive among younger age groups.

In multivariable logistic regression models, age remained the strongest factor associated with AI-based screening classification. HPV status showed an inverse association with AI screen-positive classification (OR = 0.51; 95% CI: 0.28–0.92; *p* = 0.024), indicating lower odds of screen-positive classification among participants with recorded HPV positivity. Abdominal circumference also showed an inverse association with AI screen-positive classification (OR = 0.53; 95% CI: 0.31–0.92; *p* = 0.024). These findings should not be interpreted as biologically protective effects or as evidence that these variables influenced the model output, since neither HPV status nor abdominal circumference was used as an input variable by CITOBOT AI. Rather, these associations may reflect characteristics of the study population, timing or completeness of clinical reporting, selection patterns within the screening pathway, or residual confounding. Therefore, the secondary regression analysis should be interpreted as exploratory and hypothesis-generating. Detailed results of the regression analyses are presented in Table 8.

**Table 8:** Secondary analysis: factors associated with AI-based screening classification.

Variable	Odds Ratio (OR)	95% CI	*p*-value
Age group			
17–30 years	4.85	2.37–9.93	<0.001
31–50 years	2.01	1.07–3.77	0.030
≥51 years	Reference	–	–
Hypertension	0.47	0.14–1.51	0.204
Diabetes mellitus	0.83	0.24–2.79	0.758
Cancer history	1.79	0.42–7.53	0.429
HPV status	0.51	0.28–0.92	0.024
Abdominal circumference	0.53	0.31–0.92	0.024

Odds ratios were estimated using multivariable logistic regression.

The dependent variable corresponds to the AI-based screening classification (screen-positive vs. screen-negative).

Results are presented as odds ratios (OR) with 95% confidence intervals (CI).

The analysis was conducted as a secondary, exploratory assessment following model validation, aiming to examine associations between selected non-visual clinical variables and AI-based screening classification. These results should not be interpreted as evidence of causality or statistical independence.

## Discussion

The objective of this study was to evaluate CITOBOT AI, an artificial intelligence–driven system designed to support real-world cervical cancer screening using colposcopy imaging. The results demonstrate that the model achieved strong internally validated screening performance in a real clinical context, with high levels of accuracy, sensitivity, specificity, and discriminative capacity. Together with the balanced class-specific performance and the low frequency of false positive and false negative predictions observed in the confusion matrix, these findings indicate that CITOBOT AI maintains both precision and sensitivity, which are essential characteristics for image-based cervical cancer screening tools. Overall, the results support the potential utility of CITOBOT AI as a screening support system for real-world cervical cancer screening using colposcopy imaging.

The use of a binary Risk versus No Risk classification should be interpreted considering the intended use of CITOBOT AI as a screening support tool. In this context, the model is not intended to replace histopathological diagnosis or to classify lesion severity. Rather, its purpose is to identify women who should undergo confirmatory evaluation through colposcopy and biopsy. Therefore, grouping CIN I, CIN II, CIN III, and invasive cancer within the Risk category is consistent with the screening logic of the system: a screen-positive result triggers further diagnostic assessment, while histopathology remains the reference standard for lesion grading and therapeutic decision-making.

The recent literature on artificial intelligence applied to cervical cancer presents a wide range of approaches, spanning models based on clinical and behavioral risk factors to automated systems for interpreting cytology, colposcopy, and advanced imaging modalities (26). Algorithms trained on structured clinical data have shown that machine learning can achieve high classification accuracy without using images, as demonstrated by models combining supervised learning techniques for risk prediction (19). However, these approaches do not address the fundamental challenge of interpreting cervical images at the point of care, which remains a critical bottleneck in screening programs. In contrast, CITOBOT AI directly targets this gap by focusing on colposcopy images acquired during routine clinical practice.

In parallel, advances in digital cytology have shown that artificial intelligence can accelerate processing times and reduce diagnostic workload, in some cases delivering results within seconds (27). Nevertheless, these systems rely on whole-slide cytology images obtained under highly controlled laboratory conditions, which differ substantially from the variability inherent in colposcopy images captured during routine examinations. Recent reviews have emphasized that many high-performing AI models are trained on curated datasets with limited clinical validation, restricting their generalizability to everyday practice (11, 26). In this context, the present study contributes to the existing evidence by validating an image-based AI system using colposcopy images collected in a public hospital under real-world screening conditions.

A distinctive feature of CITOBOT AI is its segmentation-plus-classification architecture. By incorporating automated segmentation of the cervix and transformation zone prior to risk stratification, the system addresses one of the main technical challenges in cervical image analysis: the presence of visual noise, artifacts, and non-cervical structures that can degrade deep learning model performance. The literature has highlighted segmentation as one of the most technically challenging steps in cytology and digital colposcopy due to cellular overlap, anatomical variability, and heterogeneous image quality (27). In this study, the integration of the Segment Anything Model enabled the algorithm to focus on anatomically relevant regions, in line with current recommendations emphasizing segmentation as a key component for improving robustness in deep learning–based cervical image analysis (20, 26). The stable training dynamics and the balanced category-specific performance observed across both risk groups provide empirical support for the effectiveness of this design.

Beyond cervical cancer screening, the methodological pipeline developed in this study may have broader applicability for image-based screening in gynecologic oncology. The combination of transfer learning, automated segmentation, and mobile deployment could potentially be adapted to other visual or image-dependent screening contexts, particularly in resource-limited settings where specialist availability, diagnostic delays, and follow-up barriers remain major challenges. However, such adaptation would require disease-specific datasets, clinical validation, and evaluation within the corresponding diagnostic pathways.

An additional contribution of this study is the secondary analysis examining the relationship between demographic and clinical variables and the AI-based risk classification. This analysis was not intended to establish clinical causality or statistical independence, but rather to explore whether AI-based screening outputs were related to selected non-visual clinical variables. The finding that age remained associated with the AI-based classification is consistent with the epidemiology of cervical lesions and the natural history of human papillomavirus infection, suggesting alignment between the model’s outputs and known population-level patterns. HPV status showed an unexpected inverse association with AI screen-positive classification. This finding should be interpreted cautiously and should not be considered biologically protective or as evidence that HPV status influenced the model output, since HPV status was not used as an input variable by CITOBOT AI. Rather, it may reflect sample-specific characteristics, timing or completeness of HPV reporting, clinical selection patterns, or residual confounding. Similar caution applies to abdominal circumference, which also showed an inverse relationship with AI screen-positive classification and should not be interpreted as a causal or biologically protective factor. Overall, this exploratory analysis provides a preliminary assessment of how selected non-visual clinical variables relate to AI-based screening classification, while model predictions remained based exclusively on image-derived features.

Beyond diagnostic performance metrics, the operational characteristics of CITOBOT AI are relevant when considering its potential applicability in low-resource settings. The system was designed as a portable and autonomous tool that does not require continuous internet connectivity. This design aligns with evidence from implementation studies indicating that women and healthcare providers value screening tools that reduce waiting times and diagnostic uncertainty, provided that confidentiality and appropriate communication are ensured (13). By enabling image capture, segmentation, and risk stratification within a single visit, CITOBOT AI may support more streamlined screening processes in settings where delays and loss to follow-up remain important challenges (6).

Despite these encouraging results, several limitations should be acknowledged. First, the study was conducted in a single healthcare center within one geographic region and included women who had undergone colposcopy and biopsy, rather than a population-based primary screening sample. This referred clinical cohort may introduce spectrum bias and may limit the applicability of the findings to broader primary screening populations with different disease prevalence, clinical profiles, and healthcare pathways. Patient-level partitioning was used to prevent data leakage between training and validation subsets; however, all analyses were performed within the same single-center clinical dataset. Although methodological safeguards were used to reduce overfitting, the absence of an external validation cohort limits conclusions regarding generalizability. Therefore, external validation using independent multicenter and population-based datasets is required to assess model performance across diverse populations, acquisition conditions, devices, and clinical workflows. Second, the present study focused on internal screening performance using diagnostic accuracy metrics, including sensitivity, specificity, predictive values, confusion matrix analysis, and AUC. Calibration metrics and decision curve analysis were not included in this internal validation study. These analyses should be incorporated into future prospective evaluations to assess probabilistic performance, threshold behavior, and clinical utility across screening decision pathways. In addition, no direct comparison with human colposcopist performance was conducted; future studies should evaluate CITOBOT AI against clinician performance and assess whether the system improves referral timeliness, diagnostic workflow, and follow-up adherence. Third, the binary screen-negative versus screen-positive endpoint was defined according to the intended use of CITOBOT AI as a screening support tool to identify women requiring confirmatory colposcopy and biopsy. This design supports the role of CITOBOT AI in distinguishing women with possible cervical abnormalities from those without lesions, rather than replacing histopathological grading or treatment decision-making. Because CITOBOT AI was trained and evaluated using a predefined binary screening endpoint, lesion-grade-specific performance metrics for CIN I, CIN II, CIN III/CIS, or carcinoma were not estimated in the present study. Future studies using larger datasets and models specifically designed for multiclass or CIN II+ classification are needed to assess grade-specific diagnostic performance. In addition, because the number of carcinoma cases was limited, lesion-grade-specific performance, particularly for invasive cancer, should be further assessed in larger datasets with greater representation of high-grade and malignant lesions. Finally, CITOBOT AI is intended to support clinical decision-making and should be used as a complement to—rather than a replacement for—clinical judgment, colposcopic assessment, and histopathological confirmation within clearly defined ethical and governance frameworks.

In conclusion, this study provides evidence that CITOBOT AI demonstrates strong internally validated screening performance for real-world cervical cancer screening using colposcopy imaging. By showing high diagnostic accuracy and balanced error profiles, the system supports the feasibility of image-based artificial intelligence as a screening support approach. Further multicenter and implementation studies are needed to assess its impact on screening workflows, follow-up adherence, and population-level outcomes in resource-limited settings.

## Data Availability

The datasets presented in this study can be found in online repositories. The names of the repository/repositories and accession number(s) can be found at: the datasets generated and analyzed during the current study are publicly available in the Pontificia Universidad Javeriana Dataverse Repository under the title Citobot AI. The data can be accessed via the following DOI: https://doi.org/10.60790/P2WJOV all data were anonymized prior to publication to ensure participant confidentiality.
